# The *Neurospora crassa* PP2A Regulatory Subunits RGB1 and B56 Are Required for Proper Growth and Development and Interact with the NDR Kinase COT1

**DOI:** 10.3389/fmicb.2017.01694

**Published:** 2017-09-05

**Authors:** Hila Shomin-Levi, Oded Yarden

**Affiliations:** Department of Plant Pathology and Microbiology, The Robert H. Smith Faculty of Agriculture, Food and Environment, The Hebrew University of Jerusalem Rehovot, Israel

**Keywords:** arthroconidia, PP2A, COT1, NDR kinase, PR55, PR61, *Neurospora crassa*

## Abstract

COT1 is the founding member of the highly conserved nuclear Dbf2-related (NDR) Ser/Thr kinase family and plays a role in the regulation of polar growth and development in *Neurospora crassa* and other fungi. Changes in COT1 phosphorylation state have been shown to affect hyphal elongation, branching, and conidiation. The function of NDR protein kinases has been shown to be regulated by type 2A protein phosphatases (PP2As). PP2As are heterotrimers comprised of a catalytic and scaffolding protein along with an interchangeable regulatory subunit involved in determining substrate specificity. Inactivation of the *N. crassa* PP2A regulatory subunits *rgb-1* and *b56* conferred severe hyphal growth defects. Partial suppression of defects observed in the *rgb-1*^RIP^ strain (but not in the Δ*b56* mutant) was observed in *cot-1* phosphomimetic mutants, demonstrating that altering COT1 phosphorylation state can bypass, at least in part, the requirement of a functional RGB1 subunit. The functional fusion proteins RGB1::GFP and B56::GFP predominantly localized to hyphal tips and septa, respectively, indicating that their primary activity is in different cellular locations. COT1 protein forms exhibited a hyperphosphorylated gel migration pattern in an *rgb-1*^RIP^ mutant background, similar to that observed when the fungus was cultured in the presence of the PP2A inhibitor cantharidin. COT1 was hypophosphorylated in a Δ*b56* mutant background, suggesting that this regulatory subunit may be involved in determining COT1 phosphorylation state, yet in an indirect manner. Reciprocal co-immunoprecipitation analyses, using tagged COT1, PPH1, RGB1, and B56 subunits established that these proteins physically interact. Taken together, our data determine the presence of a functional and physical link between PP2A and COT1 and show that two of the PP2A regulatory subunits interact with the kinase and determine COT1 phosphorylation state.

## Introduction

The ability to form polarized cells is a fundamental prerequisite for proper growth and development of any organism. Hyphae are one of the most polarized cells known. Polarized hyphal tip extension and lateral hyphal branching enable filamentous fungi to inhabit beneficial niches, forage and utilize substrates and interact with mating partners or hosts (Harris, 2006; Riquelme et al., 2011; Turra and Di Pietro, 2015). *Neurospora crassa* is among the most studied models for hyphal growth and morphogenesis (Davis, 2000; Galagan et al., 2003; Borkovich et al., 2004; Riquelme et al., 2011; Roche et al., 2014).

The nuclear DBF2-related (NDR) COT1 kinase, plays significant roles in polar growth of *N. crassa* (Yarden et al., 1992; Gorovits et al., 2000; Gorovits and Yarden, 2003; Ziv et al., 2009, 2013; Maerz and Seiler, 2010; Herold and Yarden, 2017). In addition, the GC kinase POD6 and the two COT1 co-activators, MOB2A and MOB2B, have been shown to be required for hyphal tip extension but not essential for the establishment of polarity (Seiler and Plamann, 2003; Seiler et al., 2006; Maerz et al., 2009, 2012; Ziv et al., 2013). The temperature sensitive *cot-1* mutant exhibits restricted colonial growth at the restrictive temperature, which is characterized by almost complete cessation of hyphal tip growth accompanied by hyperbranching (Steele and Trinci, 1977; Yarden et al., 1992; Gorovits et al., 2000; Ziv et al., 2009). COT1 activity is, at least in part, regulated by phosphorylation of the conserved residues Ser417 and Thr589 of this protein (Ziv et al., 2009, 2013; Maerz and Seiler, 2010; Maerz et al., 2012). Assuming that phosphorylation of these residues is reversible, protein phosphatases are also expected to play a role in functional regulation of COT1. In fact, inhibition of Ser/Thr type 2A protein phosphatase (PP2A) by the highly specific PP2A inhibitor okadaic acid (OA), was shown to increase NDR kinase phosphorylation and activity in both human and yeast cells, suggesting that NDR kinases can be inactivated by PP2A-driven dephosphorylation (Millward et al., 1999; Hergovich et al., 2005, 2006; Stegert et al., 2005).

PP2A is a highly conserved serine/threonine phosphatase in eukaryotic organisms, and plays an essential role in the regulation of numerous cellular activities (Millward et al., 1999; Janssens and Goris, 2001; Shi, 2009; Lillo et al., 2014; Sangodkar et al., 2015). PP2A has a heterotrimeric structure that consists of an A (∼65 kDa) scaffolding subunit, a C (∼35 kDa), catalytic subunit, and interchangeable B regulatory subunits (with varying molecular masses) (Xu et al., 2006; Virshup and Shenolikar, 2009). In human and yeast, the two interchangeable scaffolding subunits, PR65α and PR65β, have been shown to dimerize with the C subunit and affect PP2A catalytic activity. In addition to the A subunit, the B regulatory subunits have also been shown to modulate PP2A activity by dictating substrate specificity and subcellular localization (Millward et al., 1999; Gordon et al., 2011). In humans, four B subunit families, designated B (PR55), B′ (B56 or PR61), B″ (PR72), and B″′ (PR93/PR110 striatin), have been identified (Janssens and Goris, 2001; Xu et al., 2006; Shi, 2009; Sents et al., 2013). The existence of a fifth B subunit class has been reported in viruses (Gentry et al., 2005). Each family consists of two to five isoforms that are encoded by different genes. Overall, a major hallmark of PP2A is its multi-unit assembly and holoenzyme complex formation, which regulates its interactions with its substrates and additional (non-phosphatase) complex members (Seshacharyulu et al., 2013). Mutation of the PP2A regulatory and scaffolding subunits affect holoenzyme composition and can also interfere with or abolish PP2A activity. This has been demonstrated both in mammalian as well as fungal systems, including in *N. crassa* (Yang et al., 2004; Ruediger et al., 2011; Seshacharyulu et al., 2013).

In many eukaryotic organisms, two isoforms of the PP2A catalytic subunits have been identified (Janssens and Goris, 2001; Gentry et al., 2005; Xu et al., 2006; Shi, 2009). This is also the case in *N. crassa*, where two genes that encode the two PP2A catalytic subunits, PPH1 and PPG1 (NCU06630 and NCU06563, respectively) are known. Viable strains of *N. crassa* in which *pph-1* was inactivated could only be maintained as heterokaryons, indicating that this gene is essential for fungal growth (Yatzkan and Yarden, 1995; Yatzkan et al., 1998; Ghosh et al., 2014). Partial inactivation of the *pph1* gene by antisense expression in another filamentous fungus, *Sclerotinia sclerotiorum*, conferred drastic inhibition of fungal growth and development (Erental et al., 2007). The second PP2A catalytic subunit, PPG1, associates with the STRIPAK complex and deletion of the corresponding gene, *ppg-1*, affected cell fusion, aerial mycelium, and protoperithecia formation (Dettmann et al., 2013). The significance of STRIPAK in cell-signaling, hyphal fusion, virulence, and sexual/asexual development has also been demonstrated in other fungi (Beier et al., 2016). Thus, although PPH1 and PPG1 show 56% sequence identity, not all of their functions overlap.

In *N. crassa*, three B-family regulatory subunits (RGB1, B56, and Striatin) have been identified. RGB1 has been previously characterized and its inactivation was shown to result in slow growth, abnormal hyphal morphology, female sterility, and an abundant production of arthroconidia (Yatzkan and Yarden, 1999). Significant progress has been made in analysis of Striatin, which is part of the STRIPAK complex, including its roles in fruiting body formation and hyphal fusion (Bloemendal et al., 2012; Dettmann et al., 2013; Beier et al., 2016; Kück et al., 2016). The functional significance of the B56 subunit has not been analyzed in this fungus.

Mutations in *rgb-1* or *b56* homologs have been shown to confer drastic morphological changes in several fungal species. Deletion of the *Fusarium verticillioides rgb-1* homolog *ppr1* has been shown to alter hyphal growth and conidiation, while deletion of the *b56* homolog *ppr2* resulted in moderate growth defects, accompanied by early hyphal branching during conidial germination (Shin et al., 2013). In *Aspergillus nidulans*, both *pabA* and *parA* (homologs of *rgb-1* and *b56*, respectively) are involved in asexual and sexual development, and their deletion affected cleistothecium, ascospore, and ascus production. When crossing these mutants with the wild type strain, all progeny were able to produce a normal fruiting body, with viable ascospores. PP2A regulatory subunits are involved in self-fertilization but not in heterothallic sexual development in *A. nidulans* (Zhong et al., 2014). When RNAi-based gene silencing was used to reduce *rgb1* expression in the plant pathogen *S. sclerotiorum*, cells were viable, but exhibited slow growth. Furthermore, *rgb1* silencing affected melanin biosynthesis, sclerotial development, and pathogenesis (Erental et al., 2007).

The high sequence conservation of RGB1 and B56 across the fungal kingdom, alongside the reported abnormal hyphal elongation and branching upon inactivation of their corresponding genes, led us to hypothesize that at least some of these effects may be due to an interaction between PP2A and the *N. crassa* COT1 pathway. Here, we report on the involvement of RGB1 and B56 in regulation of hyphal growth and development in *N. crassa* and establish a functional and physical link between these subunits and the NDR kinase COT1.

## Materials and Methods

### Strains, Media, and Growth Conditions

General protocols for growth media and growth conditions for *N. crassa* have been previously described (Davis, 2000) and are also available at the Fungal Genetics Stock Center^1^. Strains used in this study are listed in **Table 1**. All strains, excluding MYC-PPH1, were grown in either liquid or solid (supplemented with 1.5% agar) Vogel’s minimal medium, with 1.5% (w/v) sucrose (VgS). The MYC-PPH1 strain was grown in liquid Vogel’s minimal medium with 0.1% glucose, supplemented with 0.17% arginine and 0.01 M quinic acid, at pH 5.8 (Cheng et al., 2001). When required, the medium was supplemented with 10 μg/ml hygromycin B (Duchefa), 100 mM cantharidin (Abcam Biochemicals) or 100 μg/ml L-histidine (Sigma–Aldrich).

**Table 1:** *Neurospora crassa* strains used in this study.

Strain	Genotype	Source
Wild type	*74-OR23-1 A*	FGSC#987
Wild type	*ORS-SL6 a*	FGSC#4200
*myc-cot-1*	*myc-cot-1*	Ziv et al., 2009
*cot-1*(S189A)	*myc-cot-1*(S189A)	Ziv et al., 2009
*cot-1*(S189E)	*myc-cot-1*(S189E)	Ziv et al., 2009
*cot-1*(S417A)	*myc-cot-1*(S417A)	Ziv et al., 2009
*cot-1*(S417E)	*myc-cot-1*(S417E)	Ziv et al., 2009
*cot-1*(T589A)	*myc-cot-1*(T589A)	Ziv et al., 2009
*cot-1*(T589E)	*myc-cot-1*(T589E)	Ziv et al., 2009
*his-3*	*his-3^-^A*	FGSC#6103
*his-3*	*his-3^-^a*	FGSC#9716
*cot-1-gfp*	*cot-1-sgfp+*	Feldman et al., 2013
*myc-pph1*	*pqa-5*; *myc-pph1*	Yang et al., 2004
*rgb-1*^RIP^; *cot-1*	*rgb-1*^RIP^; *myc-cot-1*	Stephan Seiler
Δ*b56*; *cot-1*	Δ*b56*; *myc-cot-1*	(Institute of Biology –
*rgb-1*^RIP^; *cot-1*(ts)	*rgb-1*^RIP^; *cot-1*	Molecular Plant
Δ*b56*; *cot-1*(ts)	Δ*b56*; *cot-1*	Physiology and Freiburg Institute for Advanced Studies, Germany)
*cot-1*(S189A); *rgb-1*^RIP^	*myc-cot-1*(S189A); *rgb-1*^RIP^	This study
*cot-1*(S189E); *rgb-1*^RIP^	*myc-cot-1*(S189E); *rgb-1*^RIP^	This study
*cot-1*(S417A); *rgb-1*^RIP^	*myc-cot-1*(S417A); *rgb-1*^RIP^	This study
*cot-1*(S417E); *rgb-1*^RIP^	*myc-cot-1*(S417E); *rgb-1*^RIP^	This study
*cot-1*(T589A); *rgb-1*^RIP^	*myc-cot-1*(T589A); *rgb-1*^RIP^	This study
*cot-1*(T589E); *rgb-1*^RIP^	*myc-cot-1*(T589E); *rgb-1*^RIP^	This study
*cot-1*(S189A); Δ*b56*	*myc-cot-1*(S189A); Δ*b56-hygR*	This study
*cot-1*(S189E); Δ*b56*	*myc-cot-1*(S189E); Δ*b56-hygR*	This study
*cot-1*(S417A); Δ*b56*	*myc-cot-1*(S417A); Δ*b56-hygR*	This study
*cot-1*(S417E); Δ*b56*	*myc-cot-1*(S417E); Δ*b56-hygR*	This study
*cot-1*(T589A); Δ*b56*	*myc-cot-1*(T589A); Δ*b56-hygR*	This study
*cot-1*(T589E); Δ*b56*	*myc-cot-1*(T589E); Δ*b56-hygR*	This study
*rgb-1*^RIP^; *his-3^-^*	*rgb-1*^RIP^; *his-3^-^*	This study
Δ*b56*; *his-3^-^*	Δ*b56*; *his-3^-^*	This study
*rgb-1-gfp*	*Pccg-1-rgb-1-gfp*; *his-3*	This study
*b56-gfp*	*Pccg-1-b56-gfp*; *his-3*	This study
*rgb-1-gfp*; *myc-cot-1*	*Pccg-1-rgb-1-gfp*; *myc-cot-1*	This study
*b56-gfp*; *myc-cot-1*	*Pccg-1-b56-gfp*; *myc-cot-1*	This study

### Determination of Growth Rate, Distances between Branches, Aerial Hyphae Height, and Conidiation Rate

For growth rate measurements, a 10-μl conidial suspension (2 × 10^6^ conidia/ml) was inoculated in race tubes containing 20 ml solid VgS medium, for 4 days at 34°C and growth was measured several times a day, at different intervals.

To determine the distance length between branches, glass microscope slides were covered with solid VgS medium inoculated with 10 μl of a conidial suspension (2 × 10^6^ conidia/ml) and incubated overnight at 34°C. The edges of the growing hypha were observed by light microscopy. The entire edge was documented in approximately 20 photographs and each photograph was analyzed using ImageJ 1.37 V (Rasband, W.S., U.S. National Institutes of Health, Bethesda, MD, United States). The distance between each two successive branches of leading hyphae was determined and, overall, several hundred measurements (≥200) were carried out for each colony.

For measuring aerial hyphae height, strains were grown in glass tubes containing 3 ml of solid VgS. The tubes were incubated for several days at 34°C until aerial hyphae formed and the culture matured to produce conidia.

To determine conidiation rate, strains were grown in 100 ml Erlenmeyer flasks containing 20 ml solid VgS. The flasks were incubated for 1 week at 34°C, until conidia were produced and matured. Then, the flasks were transferred to room temperature for an additional 3 days of incubation. Conidia were collected by adding 50 ml cold distilled water and vigorously shaking the flasks. The conidial suspensions were filtered through cheesecloth and subsequently centrifuged for 5 min at 4000 rpm. The supernatant was then completely removed. The conidia were then re-suspended in 5 ml cold distilled water and their number determined using a hemocytometer.

All the results presented are the average of at least three independent experiments for each strain. Statistical analysis was performed using JMP statistical software (version 7) by Student’s *t*-test. *P*-value less than 0.05 were considered significant.

### Construction of *rgb-1-gfp* and *b56-gfp* Strains

**FIGURE 1:**
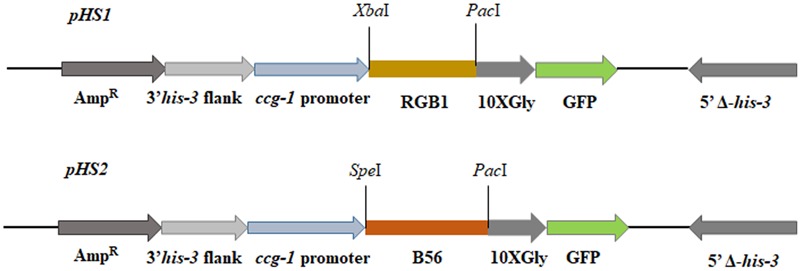
pHS1 and pHS2, designed on the basis of the C-terminal epitope tag and *gfp* expression vector pCCG::C-Gly::GFP (Honda and Selker, 2009), used for GFP-tagging of RGB1 and B56, respectively.

To tag the RGB1 and B56 proteins with a green fluorescent protein (GFP), the putative *rgb-1* and *b56* open reading frames (2087 and 2255 bp, respectively) were amplified, without the stop codon, as *Xba*I/*Pac*I (2098 bp) and *Spe*I/*Pac*I (2267 bp) fragments, respectively. The primers used for this process are listed in **Table 2**. The purified DNA amplicons were ligated into a pCCG::C-Gly::GFP vector via a flexible poly-glycine linker. The vector included the *N. crassa ccg-1* promoter and DNA fragments for targeting the construct to the *his-3* locus (Honda and Selker, 2009). Proper gene fusion was verified by sequencing of the construct junctions. The resulting plasmids, designated pHS1 (10576 bp) and pHS2 (10750 bp) (**Figure 1**), were used to transform *N. crassa* and facilitate cellular localization and immunoprecipitation of RGB1 and B56, respectively. pHS1 and pHS2 were linearized with *Ssp*I and transformed into the *rgb-1*^RIP^; *his-3^-^* and the Δ*b56*; *his-3^-^* mutants, according to a modified electroporation-based protocol (Ziv and Yarden, 2010). Briefly, 10 μl of a ∼500 ng/μl lineraized plasmid DNA solution were added to 30 μl of a fresh conidial suspension (3 × 10^9^/ml in a 1 M sorbitol solution) and maintained on ice. The mixture (40 μl conidia/DNA suspension) was transferred to a pre-chilled cuvette (0.2 cm gap) and subjected to electroporation in a Bio-Rad Gene Pulser II system set at 200 ohm resistance, 50 μF capacitance and 1.5 kV. Following electroporation, the cuvette was immediately placed on ice. Then, 360 μl of VgS were added to the cuvette and were plated in the appropriate plating medium. The transformants that exhibited a wild type phenotype were selected for their ability to grow without L-histidine. Transformant homokaryons were purified by isolation of microconidia. The presence of the RGB1-GFP and B56-GFP fusion proteins was determined by western blot analysis (Sambrook et al., 1989), using a primary mouse α-GFP antibody (Covance, Princeton, NJ, United States), followed by goat anti-mouse IgG-HRP secondary antibody (Santa Cruz Biotechnology, Inc, Santa Cruz, CA, United States). In addition, GFP expression within the cells was confirmed by fluorescence microscopy.

**Table 2:** Primers used in this study.

Primer name	Primer sequence
rgb-1-1F (*Xba*I)	5′-AGACTCTAGAATGGTGGAAACAGA-3′
rgb-1-2084R (*Pac*I)	5′-AGATTAATTAACAACGCGGAAAAGA-3′
b56-1F (*Spe*I)	5′-AGAACTAGTATGAAGCGGTTCAGCCAGAGA-3′
b56-2253R (*Pac*I)	5′-CATTTAATTAACCGACTTCTGGATGAACCTAC-3′

*Gene designations are followed by nucleotide position. “F” or “R” indicate forward and reverse primers, respectively.*

### Protein Extraction and Immunoblotting

For western blot analysis, 10 μl of a 5 × 10^6^ conidia/ml suspension were plated on a dialysis membrane (MWCO 12–14,000 Da; Medicell Membranes Ltd.), which were then placed on Petri dishes containing solid VgS medium and incubated for 19 h at 34°C. The mycelia were collected, flash-frozen in liquid nitrogen, and then suspended in lysis buffer [1 M sorbitol, 10 mM HEPES (pH 7.5), 5 mM EDTA, 5 mM EGTA, 5 mM NaF, 0.1 M KCl, 0.2% Triton X-100, and cOmplete protease inhibitor mixture (Roche Applied Science, Mannheim, Germany)]. The samples were homogenized using a miniature bead-beater (BioSpec, Bartlesville, United States). The homogenates were then mixed, by vortex, for 2 min and incubated on ice for 10 min. This procedure was repeated three times. The samples were centrifuged for 40 min at 13,500 rpm, and the supernatants were recovered. Protein concentration was determined using the Bradford protein assay kit (Bio-Rad, Hercules, CA, United States). Proteins were resolved on SDS-PAGE 8% gels or on Phos-tag^TM^ SDS-PAGE gels, according to the manufacture’s protocol (Wako Chemicals GmbH). Primary antibodies used included α-cMYC (Santa Cruz Biotechnology, Inc, Santa Cruz, CA, United States), polyclonal α-COT (Gorovits et al., 1999), and monoclonal or polyclonal α-GFP antibodies. The secondary antibodies used were either goat anti-rabbit IgG-HRP for α-COT and α-GFP or goat anti-mouse IgG-HRP for α-cMYC.

For immunoprecipitation of proteins, *N. crassa* mycelia samples, obtained from cultures grown in liquid VgS medium (5 × 10^6^ conidia/ml), were frozen in liquid nitrogen, pulverized, and suspended in lysis buffer (see above). Then, the samples were homogenized by 10 strokes of pestle A in a Dounce homogenizer. The homogenates were centrifuged for 20 min, 4500 rpm, 4°C and the supernatants were recovered into new tubes and centrifuged (13,000 rpm) one more time, for 40 min at 4°C. One microliter of cleared crude extract was incubated in a rotation device, for 2 h at 4°C, with 3–5 μl antibodies, followed by incubation with 20 μl A/G PLUS-Agarose immunoprecipitation reagent (Santa Cruz Biotechnology, Inc, Santa Cruz, CA, United States), overnight at 4°C. For GFP-tagged proteins, the purified crude extract was directly incubated in a rotation device, overnight at 4°C, with 25 μl GFP-Trap^®^ magnetic beads (ChromoTek GmbH). The beads were centrifuged (13,000 rpm) for 1 min at 4°C, and were washed three times with washing buffer (50 mM Tris pH = 7.5, 100 mM KCl, 10 mM MgCl_2_, 0.1% NP40). For co-immunoprecipitation (co-IP) experiments, the two last washes were performed with washing buffer supplemented with 0.5 M NaCl. Immunoprecipitated proteins were recovered by boiling the beads for 10 min at 70°C in NuPAGE^®^ Sample buffer (4×) and NuPAGE^®^ Sample Reducing agent (10×) (Invitrogen, Carlsbad, CA, United States) and were resolved on NuPAGE^®^ Bis-Tris 4–12% SDS-PAGE or 7% SDS-PAGE gels. The co-IP experiments were performed, at least twice, with negative and positive controls.

### Phos-tag Analysis

Conidia (10 μl of a 5 × 10^6^ conidia/ml suspension) were inoculated on a dialysis membrane placed on solid VgS medium. When required, the medium was supplemented with 320 μM cantharidin or the solvent used (DMSO) as a control, as described by Yatzkan and Yarden (1995). The dishes were incubated for 19 h at 34°C. Protein extraction was performed as described above. The protein samples were separated for 4 h on a 6% SDS-PAGE 100 μM Phos-tag gel, prepared according to the manufacturer’s instructions (Kinoshita et al., 2009). The gel was then washed twice, for 20 min each, with running buffer, supplemented with 10 mM EDTA, and once in running buffer without EDTA, for 10 min. The separated proteins were transferred to a nitrocellulose membrane according to the iBlot^®^ Dry Blotting System protocol (Thermo Fisher Scientific). α-cMYC antibodies were used to detect the tagged COT1 protein.

### Liquid Chromatography–Mass Spectrometry Protein Analysis

Tagged proteins immunoprecipitated with either α-cMYC or α-GFP antibodies were short-separated on a 4–12% SDS-PAGE (Invitrogen, Carlsbad, CA, United States) gel, in a manner that included all proteins in one lane. Gels were then stained with Coomassie Blue to visualize the proteins in order to enable excision. The immunoprecipitated proteins were digested with trypsin and analyzed by liquid chromatography–mass spectrometry (LC–MS) using a Q Exactive plus (Thermo Fisher Scientific) mass spectrometer, and identified with the Discoverer software version 1.4, using the *N. crassa* section of the NCBI-NR database and a decoy databases (in order to determine the false discovery rate) and the sequest and mascot search engines (Smoler Proteomic Research Center, Technion City, Haifa).

### Microscopy

Light microscopy was performed with a Zeiss Axioscope microscope, equipped with a Nikon DXM1200F digital camera. Standard fluorescence microscopy was performed with an EVOS FL Auto Cell Imaging System (Life Technologies). Confocal laser scanning inverted microscopy was performed using a LEICA TCS SP8 system, equipped with an argon solid-state laser. The strains were imaged with an excitation 488 nm laser line and emission at 510–530 nm. For scanning electron microscopy (SEM), samples were fixed, for 4 h, with 5% (v/v) glutaraldehyde in 0.1 M phosphate buffer, pH = 7.2. The samples were then washed five times with the same buffer and dehydrated in a series of 25–100% ethanol washes. The fixed samples were dried for 1 h in a Critical Point Dryer (Quorum K850) and gold-palladium-coated in a Quorum SC7620 Spatter coater apparatus. The microphotographs were recorded using a scanning electron microscope JEOL model, IT-100 LV. The images were taken with an accelerating voltage of 20 kV, at high vacuum mode and secondary electron image.

## Results

### Deletion of *b56* Alters Hyphal Growth, Branching, Aerial Hyphae Height, and Conidiation in *N. crassa*

**FIGURE 2:**
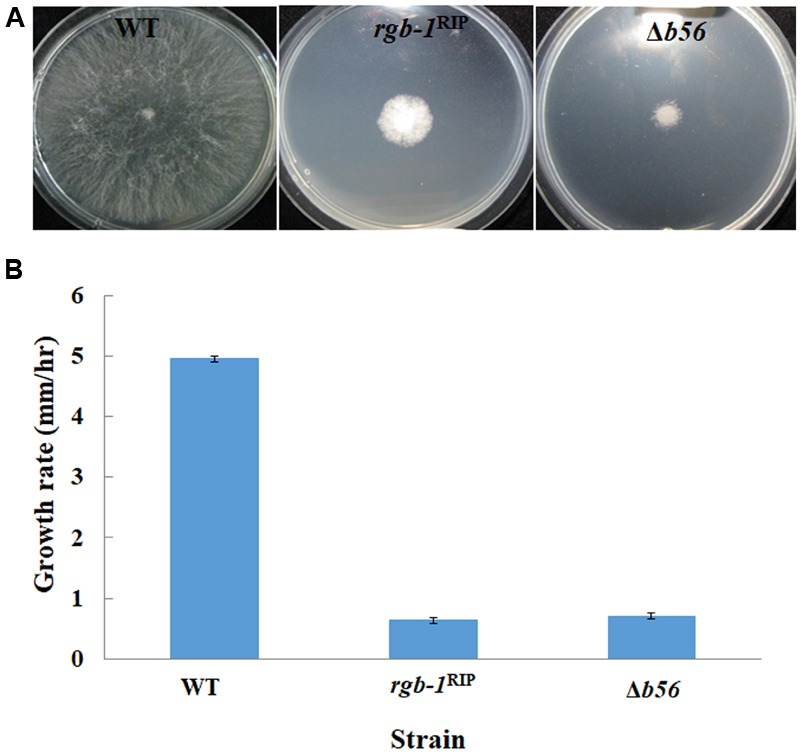
Colony morphology and growth rates of wt, *rgb-1*, and *b56* regulatory subunit-encoding gene mutants. **(A)** Colony growth of the wt, *rgb-1^RIP^*, and Δ*b56* strains grown on VgS medium at 34°C for 20 h. **(B)** Growth rate of the wt, *rgb-1^RIP^*, and Δ*b56* grown in race tubes at 34°C. Bars indicate standard error.

**FIGURE 3:**
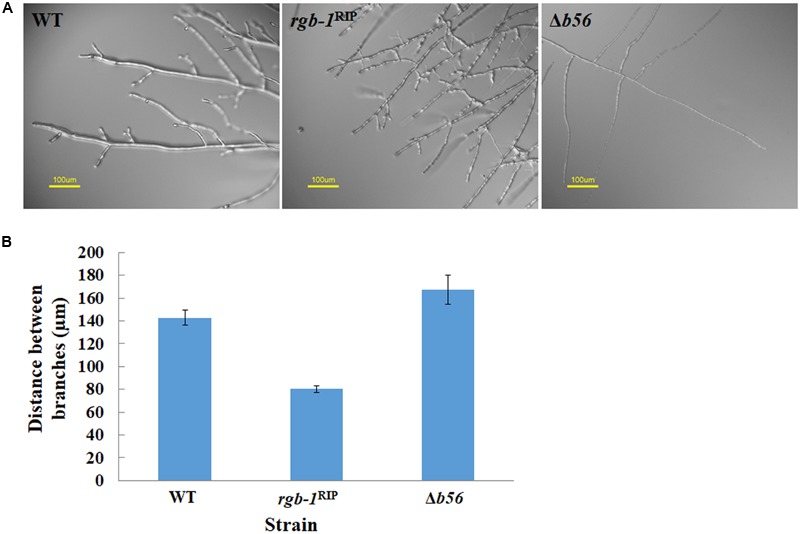
Inactivation of the PP2A regulatory subunits *rgb-1* and *b56* affects fungal morphology. **(A)** Hyphal morphology at the edge of wt, *rgb-1*^RIP^, and Δ*b56* colonies. Bar = 100 μm. **(B)** Distance between branches of the wt, *rgb-1^RIP^*, and Δ*b56* strains. Bars indicate standard error.

**FIGURE 4:**
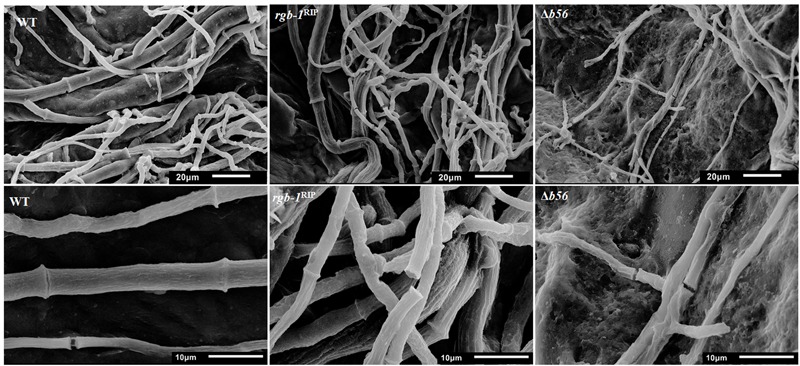
Inactivation of PP2A regulatory subunits impairs hyphal morphology. Scanning electron micrographs of wt, *rgb-1*^RIP^, and Δ*b56* hyphae from strains cultured on VgS medium. Upper panel bar = 20 μm, lower panel bar = 10 μm.

Among the three known regulatory subunits of PP2A in *N. crassa*, the function of B56 has not yet been morphologically characterized. The Δ*b56* strain, in which the gene encoding this regulatory subunit had been deleted, formed dense and compact colonies (**Figure 2A**) and grew dramatically slower (approximately 14%) than the wild type. This was similar to the growth rate (13% of that of wild type) observed for the *rgb-1*^RIP^ mutant (**Figure 2B**). Throughout all stages of asexual growth, the Δ*b56* mutant exhibited irregular mycelial morphology, which included the formation of long hyphae at later stages of growth, while *rgb-1*^RIP^ mycelium grew in a more compact and dense form (**Figure 2A**). The mutations also affected the branching distances in the leading hyphae at the periphery of the colony, with significantly shorter distances between branches in the *rgb-1*^RIP^ mutant (90 μm) and longer distances in the Δ*b56* mutant (188 μm), when compared to the wild type (143 μm). In contrast to the *rgb-1*^RIP^ mutant, where the branched hyphae appeared close to hyphal tips (apical branching), the Δ*b56* mutant branches appeared in a more distal region (lateral branching) (**Figure 3**). More detailed examination of the mutants’ hyphae by SEM analysis showed that Δ*b56* had apparently damaged, thin and wispy hyphae, with breaks along the hyphal filaments, compared to the robust mycelium of the wild type strain (**Figure 4**). The Δ*b56* strain produced only about 20% the amount of conidia produced by the wild type, yet as in the case of *rgb-1*^RIP^, they were predominantly arthroconidia.

**FIGURE 5:**
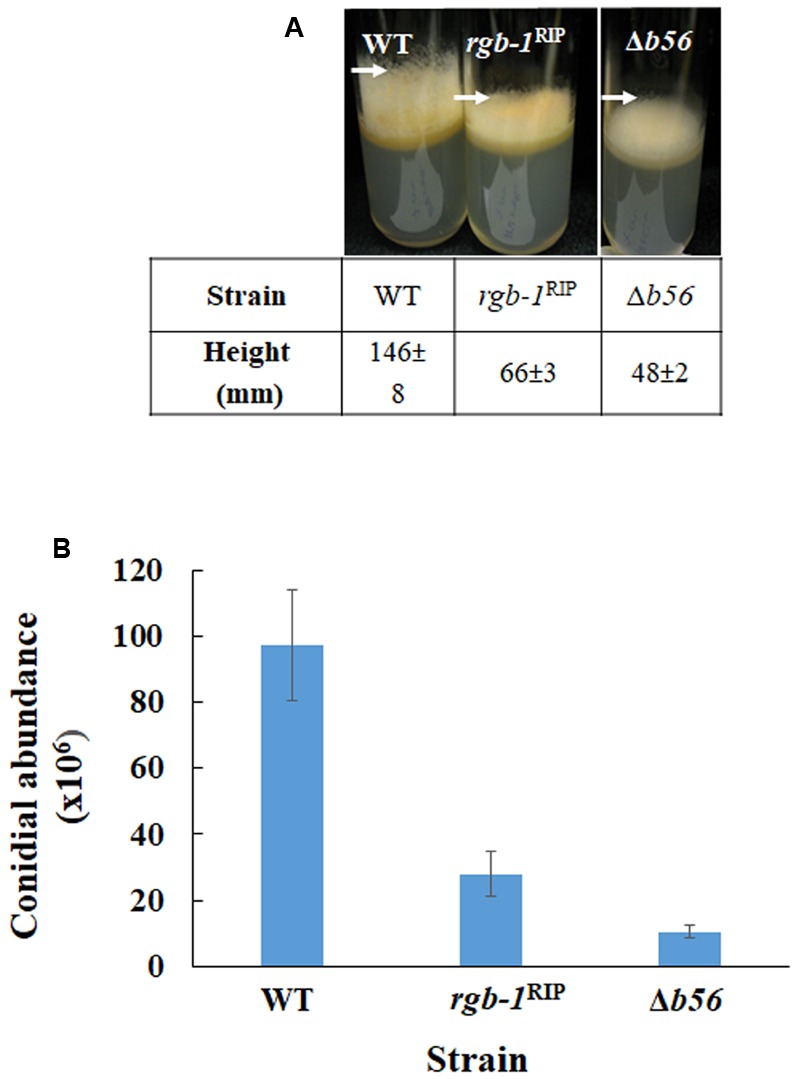
The PP2A regulatory subunits *rgb-1* and *b56* are required for proper aerial hyphae formation and conidiation. **(A)** wt, *rgb-1*^RIP^, and Δ*b56* strains grown in test tubes at 34°C for 3 days. Height of the aerial hyphae of the different strains is indicated. **(B)** Conidial abundance in the regulatory subunit mutants. Bars indicate standard error.

The Δ*b56*, as well as the previously described *rgb-1*^RIP^ strains, exhibited a defect in macroconidiation. As the production of the aerial hyphae is a prerequisite for conidiation (Springer, 1993), we examined the effect of mutations in the regulatory subunits of PP2A on formation of these structures. The height of aerial hyphae produced in the Δ*b56* and *rgb-1*^RIP^ mutants was 33 and 45% of that measured in the wild type, respectively (**Figure 5A**). This may well explain the consequent, marked, effect on conidiation that was observed: overall, the Δ*b56* and *rgb-1*^RIP^ mutants produced 22 and 28%, respectively, of the amount of conidia produced by the wild type (**Figure 5B**). However, and as mentioned above, almost of the asexual spores produced by these strains were arthroconidia. We concluded that both of these PP2A regulatory subunits are involved in the regulation of hyphal elongation, branching frequency as well as arthro- and macroconidiation in *N. crassa*.

**FIGURE 6:**
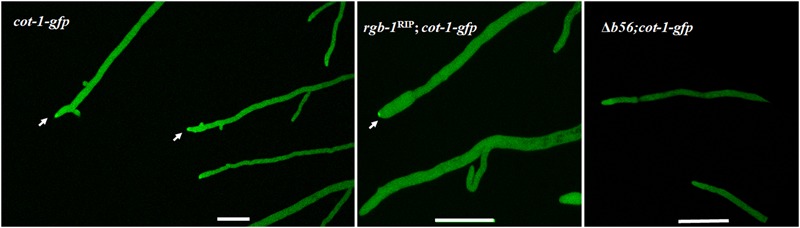
Localization pattern of COT1::GFP in *rgb-1*^RIP^ and Δ*b56* backgrounds as determine by confocal microscopy. High abundance of COT1::GFP is evident in wt and I *rgb-1*^RIP^ (marked by arrows), but not Δ*b56* backgrounds. Bars indicate 10 μm.

In light of the above findings, we examined whether these subunits affect COT1, a regulator of elongation/branching in *N. crassa*. As a first step in doing so, we examined whether inactivation of the two regulatory subunits affected COT1 localization, shown to occur within the cytoplasm, in association with plasma membrane and within the Spitzenkörper (Gorovits et al., 2000; Maerz et al., 2012; Feldman et al., 2013). To do so we generated the *rgb-1*^RIP^; *cot-1-gfp* and the Δ*b56*; *cot-1-gfp* strains (**Table 1**). While no changes in COT1 localization were observed in the *rgb-1*^RIP^ background, deletion of *b56* markedly reduced the intensity of COT1::GFP typically found in the Spitzenkörper (**Figure 6**). Nonetheless, the lack of *b56* apparently did not affect the typical presence of COT1 within the cytoplasm.

### *rgb-1* Genetically Interacts with *cot-1*

**FIGURE 7:**
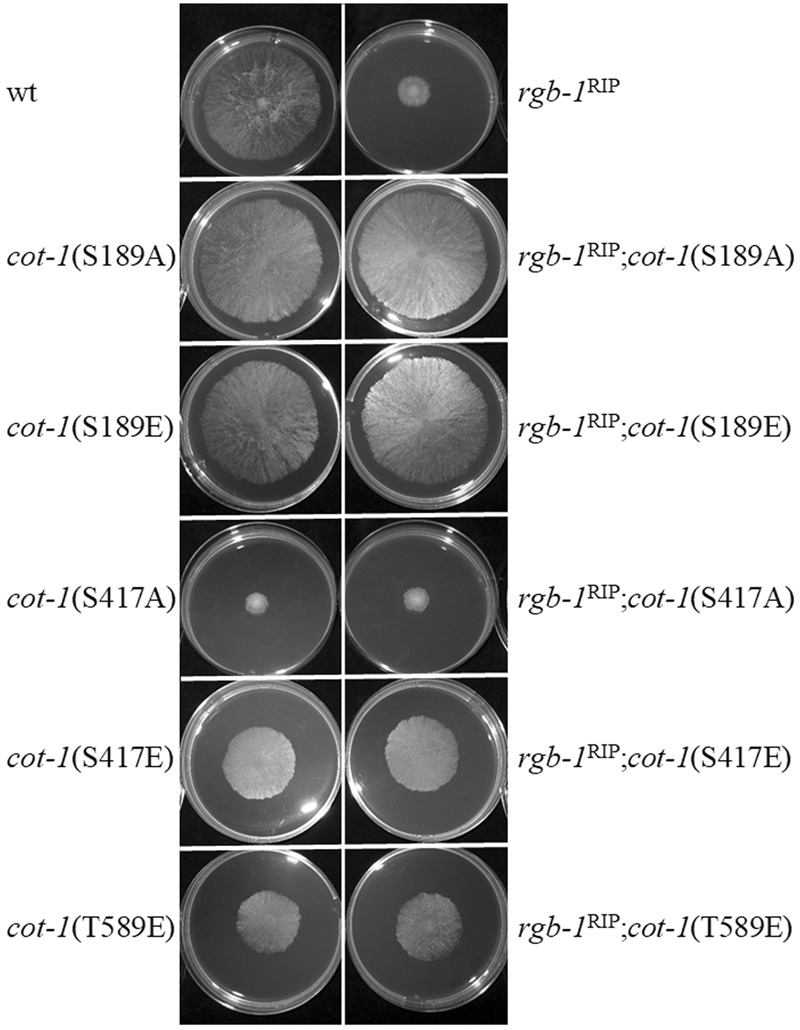
*rgb-1*^RIP^ is phenotypically suppressed in several *cot-1* phosphomimetic mutant backgrounds. Strains were cultured for 20 h at 34°C.

To further examine the possible interactions between the regulatory subunits and *cot-1*, a series of double mutants was produced and analyzed in order to determine the morphological impact of inactivation of each of the two phosphatase regulatory subunits in various *cot-1* allele backgrounds. Initial results showed that only additive effects on radial growth were noted in the *rgb-1*^RIP^; *cot-1*(ts) and Δ*b56*; *cot-1*(ts) strains grown for 44 h at permissive (25°C) or semi-restrictive (30°C) temperatures (**Table 3**). Nonetheless, we expanded the analysis to include a series of COT1 phosphomimetic mutants, in which the potentially phosphorylated residues Ser189, Ser417, and Thr589 had been substituted to glutamate or alanine, to mimic the constitutively phosphorylated and non-phosphorylated states, respectively (Ziv et al., 2009, 2013). In this case, a clear indication of a genetic interaction between *rgb-1* and *cot-1* was provided by the suppression of the slow growth phenotype of *rgb-1*^RIP^ in *cot-1*(S189A) and *cot-1*(S189E) backgrounds (**Figure 7**). The radial hyphal growth rate of these double mutants increased by 74 and 69%, respectively, compared to the parental *rgb-1*^RIP^ strain. Similar observations were recorded for *rgb-1*^RIP^ strains harboring the *cot-1*(S417E) and *cot-1*(T589E) alleles. These mutants exhibited increased hyphal growth (by 53 and 41%, respectively) as compared to *rgb-1*^RIP^ (**Figure 7**). Even though we also expected to observe phenotypic suppression of the *rgb-1*^RIP^ phenotype in the *cot-1*(S417A) and *cot-1*(T589A) backgrounds, assuming the unphosphorylated residues might bypass the requirement for the phosphatase, the growth defects evident in the *rgb-1*^RIP^ mutant were not affected, suggesting that the unphosphorylated states of COT1 residues 417 or 589 are not dependent on a functional RGB1. In contrast to the above, and despite the morphological defects observed in the Δ*b56* mutant (some of which were similar to the observed in the *rgb-1*^RIP^ strain), there was no evidence for the presence of any interaction between *b56* and *cot-1* under the experimental conditions tested.

**Table 3:** Radial growth rate of *rgb-1*^RIP^; *cot-1*(ts) and Δ*b56*; *cot-1*(ts) strains after 44 h (percent of *cot-1*(ts) growth).

Temperature	*rgb-1*^RIP^	Δ*b56*	*rgb-1*^RIP^; *cot-1*(ts)	Δ*b56*; *cot-1*(ts)
25°C (permissive temperature)	32 ± 1%	22 ± 4%	28 ± 8%	23 ± 6%
30°C (semi-restrictive temperature)	121 ± 2%	91 ± 1%	25 ± 1%	24 ± 10%

Taken together, we concluded that an epistatic interaction occurs between *cot-1* alleles and *rgb-1*, yet not with *b56* and that, in some cases, mimicking a dephosphorylated state of specific COT1 residues can bypass the requirement for the RGB1 subunit. However, this is not the case for some of the other alleles examined, as dephosphorylation of COT1 residues 417 and 589 cannot phenotypically compensate for the lack of RGB1-related phosphatase activity and hyperphosphorylation of residues 417 and 589 partially bypasses the requirement for the phosphatase. This suggests that there is more than one scenario of interactions between the NDR kinase and PP2A (see Discussion).

### RGB1 and B56 Do Not Share an Identical Cellular Distribution Pattern with COT1

**FIGURE 8:**
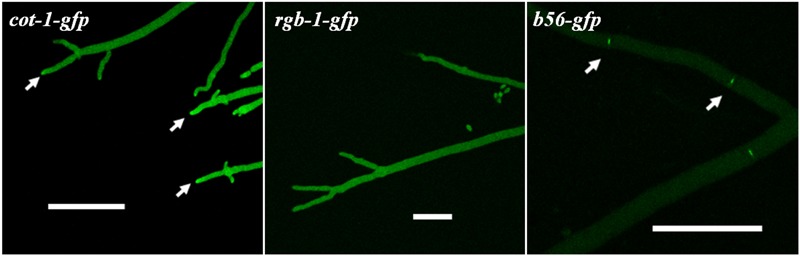
RGB1::GFP and B56::GFP predominantly localize to the hyphal tip and septa, respectively (marked by arrows), as determined by confocal microscopy. COT1 localization is presented for comparison. Bars indicate 50 μm.

In order to determine the subcellular localization of RGB1 and B56, we constructed strains expressing *rgb-1-gfp* and *b56-gfp* fusions under the control of the *ccg-1* promoter. The RGB1::GFP and B56::GFP fusion constructs (**Figure 1**) were transformed into the auxotrophic strains *rgb-1*^RIP^; *his-3^-^* and Δ*b56*; *his-3^-^*, respectively. Following the initial prototrophic selection process, we isolated transformants which exhibited wild type growth characteristics, in order to verify that the fusion proteins produced were functional RGB1 and B56 subunits. Following the use of western blot analysis to verify that the chimeric protein was properly expressed and that the fusion was of the expected size, fluorescence microscopy was used to determine the localization of the two subunits; the RGB1::GFP fusion protein localized primarily to the cytoplasm, with somewhat brighter fluorescence at the hyphal tip area, yet this was not identical to Spitzenkörper-associated localization observed in the *cot-1-gfp* strain. The highest abundance of B56::GFP was in hyphal septa, and particularly at the septal pores (**Figure 8**).

### Inactivation of *rgb-1* or *b56* Affects COT1 Phosphorylation

**FIGURE 9:**
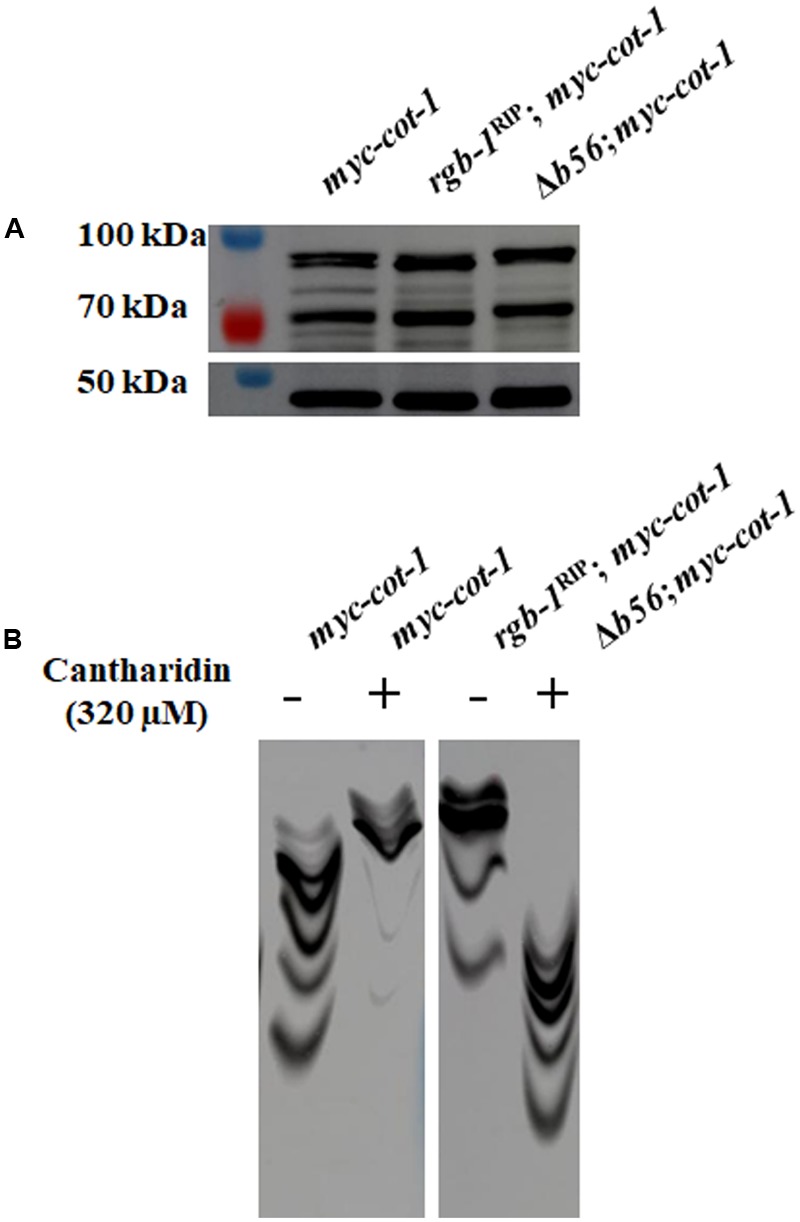
Detection of COT1 forms by western blot analysis in *rgb-1*^RIP^ and Δ*b56* backgrounds. **(A)** As resolved using a standard SDS-PAGE system. **(B)** Effect of cantharidin (left panel) or mutation of the *rgb-1* and *b56* PP2A regulatory subunits (right panels) on COT1 phosphorylation state, as evident by the resolving patterns of COT1 forms on a Mn^2+^-Phos-tag^TM^ SDS-PAGE system. MYC::COT1 was detected using anti-MYC antibodies.

We examined whether mutations in the PP2A regulatory subunits affect the phosphorylation state of COT1. First, we analyzed the effect of inactivation of the regulatory subunits on COT1 abundance. As can be seen in **Figure 9A**, no marked differences were observed in the COT1 signal in a standard SDS-PAGE gel probed with the anti-MYC antibodies. In order to also obtain a qualitative answer, concerning the potential extent of the effect on COT1 phosphorylation state, we employed the use of a Phos-tag system.

Previous reports have shown that the PP2A inhibitor cantharidin affects hyphal growth and development in *N. crassa* and *S. sclerotiorum*, by inhibiting phosphatase activity (Yatzkan and Yarden, 1995; Yatzkan et al., 1998; Erental et al., 2007). We therefore examined the influence of cantharidin on the phosphorylation state of COT1, by extracting proteins from a *myc-cot-1* strain grown in the presence or absence of the inhibitor, and resolving the proteins using Phos-tag gel electrophoresis. As seen in **Figure 9B** (left panel), in contrast to the high distribution of COT1 forms along the resolving gel lane, slower migrating (hyper-phosphorylated) COT1 protein forms were predominant in extracts obtained from cultures grown in the presence of the phosphatase inhibitor. We expanded our analysis to determine the migration patterns of phospho-COT1 in extracts obtained from the *rgb-1*^RIP^ and Δ*b56* mutants. Slower COT1 migration was detected in the *rgb-1*^RIP^ mutant background, highly mimicking the effect of cantharidin, suggesting that the inactivation of RGB1 results in hyper-phosphorylation of COT1. Unexpectedly, and although our initial screen showed no interactions between *b56* and *cot-1*, COT1 protein migration in the Phos-tag gel was faster than in the *myc-cot-1* control, suggesting that COT1 is hypophosphorylated upon inactivation of B56 (**Figure 9B**, right panel).

Taken together, these results show that PP2A impacts COT1 phosphorylation states via its RGB1 and B56 subunits, in converse manners. The RGB1 subunit may well be required for direct COT1 dephosphorylation, while we propose that B56 acts in an indirect manner, most likely, via an intermediate.

### COT1 Physically Interacts with the Catalytic and Regulatory Subunits of PP2A

**FIGURE 10:**
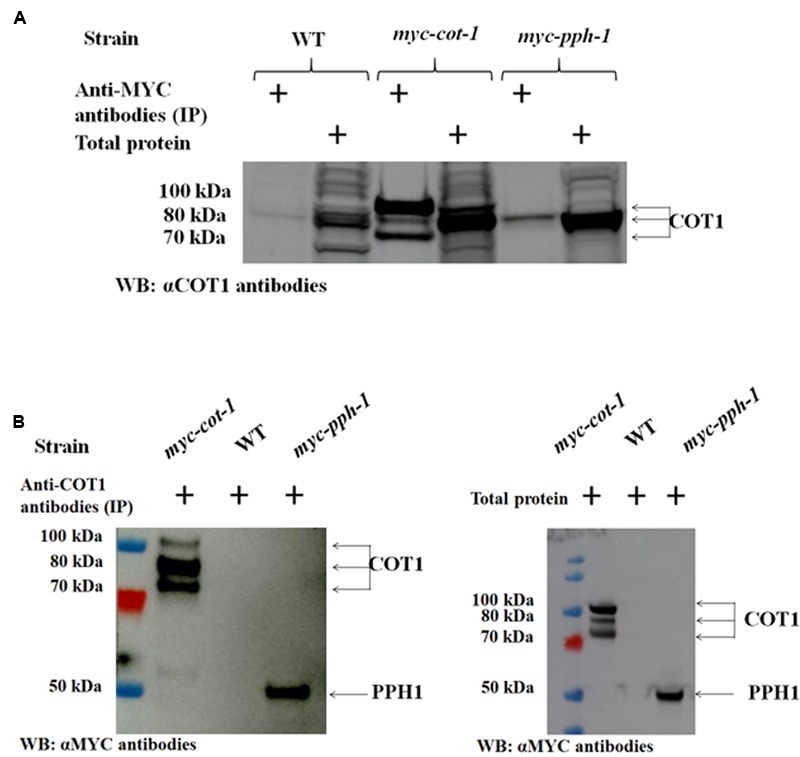
The PP2A catalytic subunit PPH1 physically interacts with COT1. **(A)** Detection of COT1 in total protein extracts and those immunoprecipitated with anti-MYC antibodies. All were probed with anti-COT1 antibodies. Arrows indicate the detected COT1 forms. **(B)** Detection of MYC::PPH1 in protein extracts immunoprecipitated with anti-COT1 antibodies and probed with anti-MYC antibodies (left panel) and in total protein extracts (right panel). Arrows indicate COT1 forms and MYC-tagged PPH1.

The function of COT1 in regulation of hyphal elongation and branching has been shown to involve, at least in part, the phosphorylation state of the kinase (Ziv et al., 2009, 2013). The fact that type 2A phosphatase function can oppose the activities of NDR kinases, has been demonstrated in mammalian cell lines (Hergovich, 2016 and references within). However, little is known regarding the physical interactions between these proteins. In order to identify potential COT1/PP2A-interactions, we conducted immunoprecipitation experiments with tagged COT1 and PP2A proteins. The presence of a physical interaction between COT1 and PPH1 was established in reciprocal co-IP experiments (**Figure 10**). Following immunoprecipitation with anti-MYC antibodies and subsequent western blot analysis using anti-COT1 antibodies, we detected a single (∼80 kDa) band corresponding to one of the COT1 forms (**Figure 10A**). For the reciprocal experiment, anti-COT1 antibodies were used for immunoprecipitation and then western blot analysis provided evidence for the presence of one band (∼50 kDa) corresponding to MYC::PPH1 (**Figure 10B**).

The physical interactions between COT1 and the PPH1 catalytic subunit was further confirmed on the basis of LC–MS analysis. Results obtained also demonstrated that additional COT1-related proteins (GUL1, PMR3, and MOB2B) physically interact with PPH1 (Supplementary Table S1).

**FIGURE 11:**
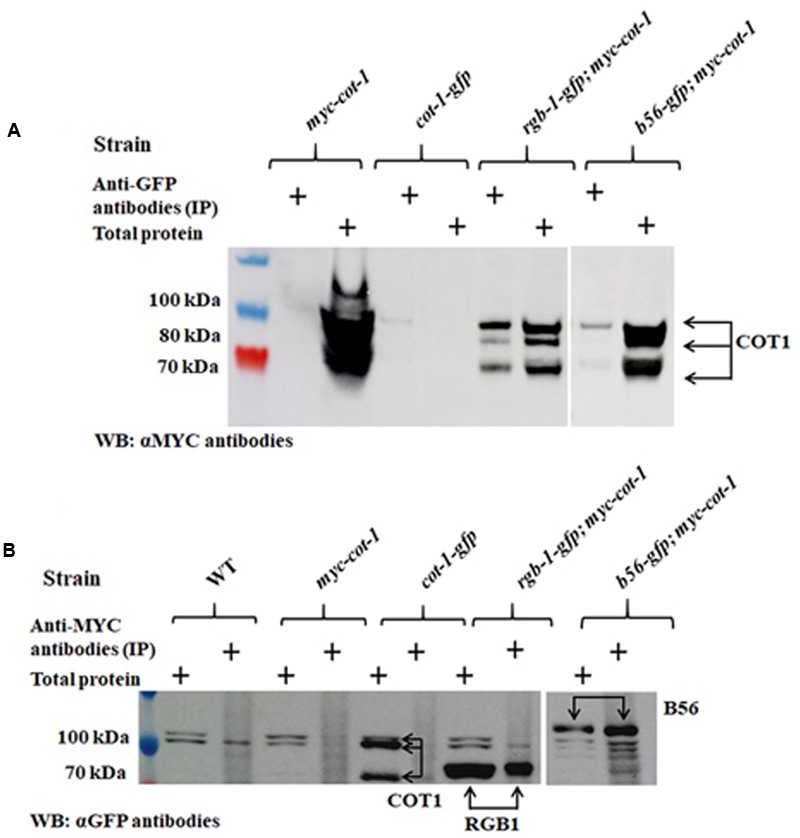
The PP2A regulatory subunits RGB1 and B56 physically interact with COT1. **(A)** Detection of COT1 in total protein extracts and those immunoprecipitated with anti-GFP antibodies and probed with anti-MYC antibodies. Arrows indicate the detected COT1 forms. **(B)** Detection of COT1 in total protein extracts and those immunoprecipitated with anti-MYC antibodies and probed with anti-GFP antibodies. Arrows indicate the detected the chimeric forms of COT1, RGB1, and B56.

We expanded the protein–protein interactions analysis to include the two PP2A regulatory subunits. To do so, we performed additional co-IP experiments with the *rgb-1-gfp* and *b56-gfp* strains that had been crossed with the *N. crassa myc-cot-1* strain. Three COT1 forms (100, 80, and 70 kDa) were detected in the crude protein extract of the *myc-cot-1* control (**Figure 11A**). These COT1 forms were also detected in the *rgb-1-gfp* immunoprecipitant demonstrating the occurrence of a physical interaction between COT1 and RGB1. The existence of physical interaction in the *rgb-1-gfp* immunoprecipitant was substantiated by detection of three COT1 forms (100, 80, and 70 kDa), as was also detected in control strains (**Figure 11A**). We also detected weakly expressed high-molecular and low-molecular weight COT1 forms (100 and 70 kDa, respectively) in the *b56-gfp*; *myc-cot-1* strain extract, indicating that a physical interaction between COT1 and the B56 subunit may also be possible. To confirm these results, we performed the reciprocal co-IP experiments using anti-MYC antibodies immunoprecipitation and then probed the proteins obtained with anti-GFP antibodies. The western blot analysis demonstrated the presence of the 80 and 100 kDa RGB1 and B56 subunit GFP protein chimeras, respectively (**Figure 11B**).

Taken together, based on the results obtained from the combination of the genetic and physical interaction analyses, we suggest that COT1 interacts with PP2A via the RGB1 subunit in a manner that affects COT1 phosphorylation state. In addition, and despite the lack of initial evidence for a genetic interaction between *b56* and *cot-1*, the co-IP experiments strongly suggest that COT1 and B56 do, in fact, also physically interact.

## Discussion

The NDR kinase COT1 and the phosphatase PP2A have both been demonstrated to be involved in various cellular processes, including cell growth and morphology, development, and signal transduction. Impairing the function of any of these components leads to significant morphological consequences affecting asexual growth and development in *N. crassa*.

As the significance of COT1 phosphorylation states with regard to hyphal elongation and branching has been clearly demonstrated (Ziv et al., 2009, 2013), it is highly conceivable that an opposing phosphatase is involved in regulation of COT1 phosphorylation in a direct manner and via other protein intermediates. Given the evidence for its interaction with other NDR kinases, PP2A is a likely candidate for this role (Hergovich, 2016). In addition to the two genes encoding the *N. crassa* PP2A catalytic subunits (*pph-1* and *ppg-1*), three known genes encoding for PP2A regulatory subunits have been identified in this fungus: *rgb-1, b56*, and *ham-3* (Striatin). As the latter, HAM3, has been shown to physically interact with PPG1 (Dettmann et al., 2013), rather than PPH1, in this report, we focused on the RGB1 and B56 PP2A subunits and their potential interactions with COT1 and involvement in hyphal development.

Previously, Yatzkan and Yarden (1999) demonstrated that inactivation of *rgb-1* by repeat induced point mutations (RIP) affected hyphal growth and conidiation. The *rgb-1*^RIP^ mutant exhibited slow hyphal growth, short distances between branches, and a reduction in the amount of conidia produced. *N. crassa* produces three types of conidia via three different sporulation pathways: macroconidia that develop from aerial hyphae, microconidia, which are smaller, uninuclear conidia, that develop directly from hyphae, usually during stress conditions, and arthroconidia, which develop from hyphal segments, especially when macroconidia and microconidia formation pathways are blocked (Springer, 1993). Inactivation of *rgb-1* resulted in the accumulation of large quantities of arthroconidia, and was suggested to have affected aerial hyphae production, possibly by blocking macroconidia and microconidia development. Here, we report that deletion of *b56* altered hyphal growth, branching, aerial hyphae height, and conidiation in the fungus. This was characterized by slow growth rate and increased distances between branches. Interestingly, the predominant conidia produced by the Δ*b56* strain were arthroconidia, in a manner highly resembling that observed in the *rgb-1*^RIP^ strain. This phenomenon resembles that also observed in the *cot-1*(T589E) mutant, suggesting a need for the phosphorylated COT1 form in regulation of aerial hyphae formation and conidiation (Ziv et al., 2009). Deletion of *dbf-2*, which encodes another NDR kinase in *N. crassa*, also conferred heavy defects in aerial hyphal formation, and increased arthroconidia and microconidia production (Dvash et al., 2010). The repeated occurrence of involvement of NDR kinases and the PP2A subunits RGB1 and B56 in arthroconidiation suggest a common key role for these components in normal asexual reproduction in *N. crassa*.

The significance of the PP2A subunits in fungal development has also been reported in other fungal species: deletion mutants of PPR1 and PPR2, the *F. verticillioides* homologs of RGB1 and B56, respectively, grew at rates of approximately 10–14 and 50% of those observed in the wild type, respectively (Shin et al., 2013). Close to complete abolishment of conidiation, accompanied by defective conidiophore morphology, were observed in the *A. nidulans* RGB1 and B56 homologs, Δ*pabA* and Δ*parA*, respectively (Zhong et al., 2014).

Protein localization can be regulated by phosphorylation, leading to changes in protein stability and translocation capacities. This has been also demonstrated in the case of COT1. Substituting COT1 T589 with Ala, mimicking an unphosphorylated COT1 589 residue state, was associated with its altered localization in the apex-associated crescent (Maerz et al., 2012). In contrast, Glu substitutions of COT1 residues S417 and T589, mimicking a constitutively phosphorylated state, did not alter COT1 localization in the hyphae. When we examined COT1 localization in the PP2A regulatory subunit mutants, we found that the kinase was present throughout the cytoplasm in both mutant backgrounds, but its characteristically punctate Spitzenkörper-association presence in the apical zone was limited in the Δ*b56* mutant. No such differences were observed in the *rgb-1*^RIP^ strain. Taken together, phosphorylation state affects COT1 localization and our results suggest that B56 plays a role in this process.

We hypothesized that the genetic interactions between *cot-1* and *rgb-1* or *b56* would be evident in strains in which the *cot-1* phosphomutants encode unphosphorylated COT1 at one of three phosphoresidues (S189A, S417A, and T589A), while the hyperphosphorylated state of COT1 would mimic the absence of RGB1 or B56, resulting in a defective phenotype similar to that of the *rgb-1* or *b56* mutants. On the basis of the phenotypic analysis of *cot-1* alleles with the *rgb-1* and *b56* mutants, we observed an epistatic interaction only in the case of *rgb-1*. Surprisingly, this interaction was observed predominantly in the hyperphosphorylated COT1 phosphomimetic strain backgrounds, rather than the constitutively unphosphorylated ones. Specifically, the *rgb-1*^RIP^ phenotype was suppressed in *cot-1*(S189A/E), cot*-1*(S417E), and *cot-1*(T589E), but not by *cot-1*(S417A) and *cot-1*(T589A) backgrounds (**Figure 7**). These results suggest that (i) dephosphorylation of COT1 residues 417 and 589 cannot phenotypically compensate for the lack of RGB1-related phosphatase activity, (ii) hyperphosphorylation of residues 417 and 589 partially bypasses the requirement for the phosphatase. One explanation for this may be the intraprotein effects of COT1 residue phosphorylation state, as reported by Maerz et al. (2012), who suggested, for example, that the presence of the *cot-1*(S417A, T589E) allele results in an instable protein conferring a drastic reduction in hyphal growth. Additional evidence concerning the various interdependent significance of various phosphorylation states of NDR kinase residues has been previously reported (Tamaskovic et al., 2003; Stegert et al., 2005; Ziv et al., 2009). Furthermore, based on the prediction that COT1 has almost 50 additional Ser and Thr potential phosphorylation sites (based on NetPhos3.1 server software analysis) that have not been experimentally analyzed to date, additional degrees of complexity with regard to the phosphorylation state of various residues in the protein may eventually become evident. It is likely that the overall combination of different phosphorylation states of the various residues, and not necessarily a single event, determines NDR kinase function. Nonetheless, and regardless of which of the residues tested involves RGB1 function, our data show that COT1 is hyperphosphorylated in the absence of this PP2A subunit (see below).

During the first stage of this study there was no evidence of a genetic interaction between *cot-1* and *b56*. Despite the morphological changes in the growth and development of Δ*b56*, its phenotype was not suppressed by any of the tested *cot-1* phosphomimetic mutants, under the limited culturing conditions tested. However, evidence for an interaction between B56 and COT1 has now been provided, based on the phosphorylation state of COT1 in a Δ*b56* background and via the presence of a physical interaction between the proteins. Hence, it is feasible that under other conditions, or with other mutants in which other phosphorylation sites had been altered, genetic interactions may become more evident.

The PP2A regulatory subunits can dictate the localization of the holoenzyme in the cell, and this localization depends on substrate specificity (Zolnierowicz, 2000; Zhong et al., 2014). Thus, various PP2A-AC dimer combinations with various regulatory subunits can be found throughout the cell. For example, the PP2A-PR55/B complex is found in the cytoskeleton, while PP2A-PR61/B′ is found in the nucleus (Janssens and Goris, 2001). Our results demonstrate that RGB1 and B56 are predominantly found in different cellular locations in *N. crassa*. In *F. verticillioides*, the RGB1 and B56 homologs were proposed to localize to the cytoplasm and septa, respectively (Shin et al., 2013), while in *A. nidulans*, both subunit homologs we detected in the septum, where they have been suggested to counteract each other’s function (Zhong et al., 2014). While RGB1 and COT1 share, at least in part, punctate localization at the hyphal tip, B56 was found to concentrate mainly at the septal pores. The fact that COT1 can also be found in the cytoplasm and along the plasma membrane (Gorovits et al., 2000) does not rule out a possible physical interaction between COT1 and B56. It may well be that the cellular distribution of B56 exceeds that which we have observed with the GFP tag and that the actual interactions occur in locations where the proteins are less abundant, as reflected in our co-IP experiments (**Figure 11**).

We incorporated the use of the Mn^2+^-Phos-tag SDS-PAGE system (Kinoshita et al., 2009) to detect mobility shifts of COT1 phosphoprotein isotypes under various conditions. Inhibition of PP2A by cantharidin, a specific PP2A inhibitor, led to the accumulation of phosphorylated COT1 forms (**Figure 9B**). This is in line with previous reports demonstrating that inhibition of NDR kinases by another PP2A inhibitor, OA, affects kinase activity, implying that NDR kinases may be substrates of PP2A (Millward et al., 1999; Hergovich et al., 2006; Fuller et al., 2008). To determine if and which of the phosphatase regulatory subunits may be involved in COT1 phosphorylation state, we monitored the COT1 phosphoprotein isotype patterns in strains in which each of the regulatory subunits had been inactivated. Our data demonstrated that COT1 is hyperphosphorylated in the *rgb-1*^RIP^ mutant, thus confirming the involvement of RGB1 in regulation of COT1 phosphorylation (**Figure 9B**). Moreover, despite the absence of any observable genetic interaction between *cot-1* and *b56* under the growth conditions tested, COT1 was hypophosphorylated in the *b56* null mutant background (**Figure 9B**, right panel). These results support the conclusion that a functional B56 subunit is required for normal COT1 phosphorylation. At the same time, the fact that the COT1 phosphoprotein isotype pattern appeared to be the result of a hypophosphorylation event, suggests that B56-mediated dephosphorylation most likely occurs on an intermediate/COT1-interacting protein(s). It is tempting to speculate that potential candidate B56 substrates may include HYM1, POD6, and MOB2A/B, who function as COT1 activators (Dettmann et al., 2012; Ziv et al., 2013).

The interaction between COT1 and PP2A was also demonstrated on the basis of physical interactions. Specifically, we have shown that PPH1, the catalytic subunit of PP2A, and the 80 kDa form of COT1 physically interact (**Figure 10**). Post-translational modifications affecting protein conformation, size and mobility may have affected the abundance of other COT1 forms, as has been previously observed (Feldman et al., 2013; Ziv et al., 2013). This interaction was further confirmed by mass spectrometry, which showed that PPH1 and COT1 can co-immunoprecipitate (Supplementary Table S1). Additional experiments demonstrated the presence of a physical interaction between the RGB1 and B56 regulatory subunits and COT1 (**Figure 11** and Supplementary Table S1). This physical interaction between B56 and COT1 was not expected, based on the lack of a genetic interaction and the hypophosphorylated COT1 protein pattern in the Δ*b56* background. A possible explanation for the presence of B56 in the immunoprecipitant could be via a COT1-B56-interacting protein(s) within the isolated COT1 complex. Taken together, and in spite of several studies that have shown the involvement of PP2A in the regulation of NDR kinases, both *in vivo* and *in vitro*, by use of the PP2A inhibitor OA (Millward et al., 1999; Tamaskovic et al., 2003; Hergovich et al., 2005, 2006; Stegert et al., 2005; Fuller et al., 2008), this is the first instance where evidence for the actual physical interaction between the two proteins has been provided.

The possibility of different PP2A regulatory subunits binding to the same protein substrate has first been described in a viral system (Cegielska et al., 1994). The PR55 regulatory subunit has been demonstrated to dephosphorylate the SV40 T-antigen Thr124 residue, resulting in SV40 inactivation. A second PP2A regulatory subunit, PR72, was shown to mediate the dephosphorylation of Ser120 and 123, resulting in activation of this protein. This phenomenon has also been demonstrated to occur in eukaryotics, where different PP2A regulatory subunits were shown to interact with death-associated protein kinase (Widau et al., 2010) and the tyrosine phosphatase DUSP19 (St-Denis et al., 2016). Thus, it appears that evidence supporting the possibility that substrate specificity conferred by the regulatory subunits of PP2A does not rule out interactions of two such subunits with a single protein, is accumulating. To what extent regulatory subunits are functionally interchangeable as mediators of *in vivo* dephosphorylation of the same residue requires further study.

The current study supports the possibility that PP2A, via its RGB1 and B56 regulatory subunits, involves both activation and inactivation of COT1 via its action on different COT1 residues. Other COT1 complex proteins may well be affected by the phosphatase and/or be involved in the PP2A–COT1 interactions. Given the structural and functional conservation of PP2A and NDR kinases it is likely that the findings reported here are relevant to additional fungi and other eukaryotes.

## Author Contributions

HS-L and OY designed the experiments. HS-L performed the experiments. HS-L and OY wrote the manuscript.

## Conflict of Interest Statement

The authors declare that the research was conducted in the absence of any commercial or financial relationships that could be construed as a potential conflict of interest.

## Footnotes

1 http://www.fgsc.net/Neurospora/NeurosporaProtocolGuide.htm

## References

[B1] Beier A. Teichert I. Krisp C. Wolters D. A. Kück U. (2016). Catalytic subunit 1 of protein phosphatase 2A is a subunit of the STRIPAK complex and governs fungal sexual development. *mBio* 7 1–11. 10.1128/mBio.00870-16 PMC4916389 27329756

[B2] Bloemendal S. Bernhards Y. Bartho K. Dettmann A. Voigt O. Teichert I. (2012). A homologue of the human STRIPAK complex controls sexual development in fungi. *Mol. Microbiol.* 82 310–323. 10.1111/j.1365-2958.2012.08024.x 22375702

[B3] Borkovich K. A. Alex L. A. Yarden O. Freitag M. Turner G. E. Read N. D. (2004). Lessons from the genome sequence of *Neurospora crassa*: tracing the path from genomic blueprint to multicellular organism. *Microbiol. Mol. Biol. Rev.* 68 1–108. 10.1128/MMBR.68.1.1-108.2004 15007097 PMC362109

[B4] Cegielska A. Shaffer S. Derua R. Goris J. Virshup D. M. (1994). Different oligomeric forms of protein phosphatase 2A activate and inhibit simian virus 40 DNA replication. *Mol. Cell. Biol.* 14 4616–4623. 10.1128/MCB.14.7.4616 8007966 PMC358834

[B5] Cheng P. Yang Y. H. Liu Y. (2001). Interlocked feedback loops contribute to the robustness of the *Neurospora* circadian clock. *Proc. Natl. Acad. Sci. U.S.A.* 98 7408–7413. 10.1073/pnas.121170298 11416214 PMC34682

[B6] Davis R. H. (2000). *Neurospora: Contributions of a Model Organism*. Oxford: Oxford University Press.

[B7] Dettmann A. Heilig Y. Ludwig S. Schmitt K. Illgen J. Fleissner A. (2013). HAM-2 and HAM-3 are central for the assembly of the *Neurospora* STRIPAK complex at the nuclear envelope and regulate nuclear accumulation of the MAP kinase MAK-1 in a MAK-2-dependent manner. *Mol. Microbiol.* 90 796–812. 10.1111/mmi.12399 24028079

[B8] Dettmann A. Illgen J. Maerz S. Schuerg T. Fleissner A. Seiler S. (2012). The NDR kinase scaffold HYM1/MO25 is essential for MAK2 MAP kinase signaling in *Neurospora crassa*. *PLoS Genet.* 8:e1002950 10.1371/journal.pgen.1002950 PMC3447951 23028357

[B9] Dvash E. Kra-Oz G. Ziv C. Carmeli S. Yarden O. (2010). The NDR kinase DBF-2 is involved in regulation of mitosis, conidial development, and glycogen metabolism in *Neurospora crassa*. *Eukaryot. Cell* 9 502–513. 10.1128/EC.00230-09 19966031 PMC2863412

[B10] Erental A. Harel A. Yarden O. (2007). Type 2A phosphoprotein phosphatase is required for asexual development and pathogenesis of *Sclerotinia sclerotiorum*. *Mol. Plant Microbe Interact.* 20 944–954. 10.1094/MPMI-20-8-0944 17722698

[B11] Feldman D. Ziv C. Gorovits R. Efrat M. Yarden O. (2013). *Neurospora crassa* protein arginine methyl transferases are involved in growth and development and interact with the NDR kinase COT1. *PLoS ONE* 8:e80756 10.1371/journal.pone.0080756 PMC3834314 24260473

[B12] Fuller S. J. Pikkarainen S. Tham E. L. Cullingford T. E. Molkentin J. D. Cornils H. (2008). Nuclear Dbf2-related protein kinases (NDRs) in isolated cardiac myocytes and the myocardium: activation by cellular stresses and by phosphoprotein serine-/threonine-phosphatase inhibitors. *Cell. Signal.* 20 1564–1577. 10.1016/j.cellsig.2008.04.013 18555663

[B13] Galagan J. E. Calvo S. E. Borkovich K. A. Selker E. U. Read N. D. Jaffe D. (2003). The genome sequence of the filamentous fungus *Neurospora crassa*. *Nature* 422 859–868. 10.1038/nature01554 12712197

[B14] Gentry M. S. Li Y. K. Wei H. J. Syed F. F. Patel S. H. Hallberg R. L. (2005). A novel assay for protein phosphatase 2A (PP2A) complexes in vivo reveals differential effects of covalent modifications on different Saccharomyces cerevisiae PP2A heterotrimers. *Eukaryot. Cell* 4 1029–1040. 10.1128/EC.4.6.1029-1040.2005 15947195 PMC1151991

[B15] Ghosh A. Servin J. A. Park G. Borkovich K. A. (2014). Global analysis of serine/threonine and tyrosine protein phosphatase catalytic subunit genes in *Neurospora crassa* reveals interplay between phosphatases and the p38 mitogen-activated protein kinase. *G3 (Bethesda)* 4 349–365. 10.1534/g3.113.008813 24347630 PMC3931568

[B16] Gordon J. Hwang J. Carrier K. J. Jones C. A. Kern Q. L. Moreno C. S. (2011). Protein phosphatase 2a (PP2A) binds within the oligomerization domain of striatin and regulates the phosphorylation and activation of the mammalian Ste20-Like kinase Mst3. *BMC Biochem.* 12:54 10.1186/1471-2091-12-54 PMC3217859 21985334

[B17] Gorovits R. Propheta O. Kolot M. Dombradi V. Yarden O. (1999). A mutation within the catalytic domain of COT1 kinase confers changes in the presence of two COT1 isoforms and in Ser/Thr protein kinase and phosphatase activities in *Neurospora crassa*. *Fungal Genet. Biol.* 27 264–274. 10.1006/fgbi.1999.1152 10441452

[B18] Gorovits R. Sjollema K. A. Sietsma J. H. Yarden O. (2000). Cellular distribution of COT1 kinase in *Neurospora crassa*. *Fungal Genet. Biol.* 30 63–70. 10.1006/fgbi.2000.1198 10955908

[B19] Gorovits R. Yarden O. (2003). Environmental suppression of *Neurospora crassa cot-1* hyperbranching: a link between COT1 kinase and stress sensing. *Eukaryot. Cell* 2 699–707. 10.1128/EC.2.4.699-707.2003 12912889 PMC178343

[B20] Harris S. D. (2006). Cell polarity in filamentous fungi: shaping the mold. *Int. Rev. Cytol.* 251 41–77. 10.1016/S0074-7696(06)51002-2 16939777

[B21] Hergovich A. (2016). The roles of NDR protein kinases in Hippo signalling. *Genes* 7:E21 10.3390/genes7050021 PMC4880841 27213455

[B22] Hergovich A. Bichsel S. J. Hemmings B. A. (2005). Human NDR kinases are rapidly activated by MOB proteins through recruitment to the plasma membrane and phosphorylation. *Mol. Cell. Biol.* 25 8259–8272. 10.1128/MCB.25.18.8259-8272.2005 16135814 PMC1234321

[B23] Hergovich A. Stegert M. R. Schmitz D. Hemmings B. A. (2006). NDR kinases regulate essential cell processes from yeast to humans. *Nat. Rev. Mol. Cell. Biol.* 7 253–264. 10.1038/nrm1891 16607288

[B24] Herold I. Yarden O. (2017). Regulation of *Neurospora crassa* cell wall remodeling via the cot-1 pathway is mediated by *gul-1*. *Curr. Genet.* 63 145–159. 10.1007/s00294-016-0625-z 27363849

[B25] Honda S. Selker E. U. (2009). Tools for fungal proteomics: multifunctional *Neurospora* vectors for gene replacement, protein expression and protein purification. *Genetics* 182 11–23. 10.1534/genetics.108.098707 19171944 PMC2674810

[B26] Janssens V. Goris J. (2001). Protein phosphatase 2A: a highly regulated family of serine/threonine phosphatases implicated in cell growth and signalling. *Biochem. J.* 353 417–439. 10.1042/bj3530417 11171037 PMC1221586

[B27] Kinoshita E. Kinoshita-Kikuta E. Koike T. (2009). Separation and detection of large phosphoproteins using Phos-tag SDS-PAGE. *Nat. Protoc.* 4 1513–1521. 10.1038/nprot.2009.154 19798084

[B28] Kück U. Beier A. M. Teichert I. (2016). The composition and function of the striatin-interacting phosphatases and kinases (STRIPAK) complex in fungi. *Fung. Genet. Biol.* 90 31–38. 10.1016/j.fgb.2015.10.001 26439752

[B29] Lillo C. Kataya A. R. A. Heidari B. Creighton M. T. Nemie-Feyissa D. Ginbot Z. (2014). Protein phosphatases PP2A, PP4 and PP6: mediators and regulators in development and responses to environmental cues. *Plant Cell Environ.* 37 2631–2648. 10.1111/pce.12364 24810976

[B30] Maerz S. Dettmann A. Seiler S. (2012). Hydrophobic motif phosphorylation coordinates activity and polar localization of the *Neurospora crassa* nuclear Dbf2-related kinase COT1. *Molecul. Cell. Biol.* 32 2083–2098. 10.1128/MCB.06263-11 PMC3372237 22451488

[B31] Maerz S. Dettmann A. Ziv C. Liu Y. Valerius O. Yarden O. (2009). Two NDR kinase-MOB complexes function as distinct modules during septum formation and tip extension in *Neurospora crassa*. *Mol. Microbiol.* 74 707–723. 10.1111/j.1365-2958.2009.06896.x 19788544 PMC4617822

[B32] Maerz S. Seiler S. (2010). Tales of RAM and MOR: NDR kinase signaling in fungal morphogenesis. *Curr. Opin. Microbiol.* 13 663–671. 10.1016/j.mib.2010.08.010 20869909

[B33] Millward T. A. Hess D. Hemmings B. A. (1999). Ndr protein kinase is regulated by phosphorylation on two conserved sequence motifs. *J. Biol. Chem.* 274 33847–33850. 10.1074/jbc.274.48.33847 10567341

[B34] Riquelme M. Yarden O. Bartnicki-Garcia S. Bowman B. Castro-Longoria E. Free S. J. (2011). Architecture and development of the *Neurospora crassa* hypha - a model cell for polarized growth. *Fungal Biol.* 115 446–474. 10.1016/j.funbio.2011.02.008 21640311

[B35] Roche C. M. Loros J. L. McCluskey K. Glass N. L. (2014). *Neurospora crassa*: looking back and looking forward at a model microbe. *Am. J. Bot.* 101 2022–2035. 10.3732/ajb.1400377 25480699

[B36] Ruediger R. Ruiz J. Walter J. (2011). Human cancer-associated mutations in the Aα subunit of protein phosphatase 2A increase lung cancer incidence in Aα knock-in and knockout mice. *Mol. Cell. Biol.* 13 3832–3844. 10.1128/MCB.05744-11 PMC3165721 21791616

[B37] Sambrook J. Fritsch E. F. Maniatis T. (1989). *Molecular Cloning: A Laboratory Manual*. Cold Spring Harbor, NY: CSH Laboratory Press.

[B38] Sangodkar J. Farrington C. C. McClinch K. Galsky M. D. Kastrinsky D. B. Narla G. (2015). All roads lead to PP2A: exploiting the therapeutic potential of this phosphatase. *FEBS J.* 283 1004–1024. 10.1111/febs.13573 26507691 PMC4803620

[B39] Seiler S. Plamann M. (2003). The genetic basis of cellular morphogenesis in the filamentous fungus *Neurospora crassa*. *Mol. Biol. Cell* 14 4352–4364. 10.1091/mbc.E02-07-0433 12960438 PMC266756

[B40] Seiler S. Vogt N. Ziv C. Gorovits R. Yarden O. (2006). The STE20/germinal center kinase POD6 interacts with the NDR kinase COT1 and is involved in polar tip extension in *Neurospora crassa*. *Mol. Biol. Cell* 17 4080–4092. 10.1091/mbc.E06-01-0072 16822837 PMC1593175

[B41] Sents W. Ivanova E. Lambrecht C. Haesen D. Janssens V. (2013). The biogenesis of active protein phosphatase 2A holoenzymes: a tightly regulated process creating phosphatase specificity. *FEBS J.* 280 644–661. 10.1111/j.1742-4658.2012.08579.x 22443683

[B42] Seshacharyulu P. Pandey P. Datta K. Batra S. K. (2013). Phosphatase: PP2A structural importance, regulation and its aberrant expression in cancer. *Cancer Lett.* 335 1–22. 10.1016/j.canlet.2013.02.036 23454242 PMC3665613

[B43] Shi Y. (2009). Serine/threonine phosphatases: mechanism through structure. *Cell* 139 468–484. 10.1016/j.cell.2009.10.006 19879837

[B44] Shin J. H. Kim J. E. Malapi-Wight M. Choi Y. E. Shaw B. D. Shim W. B. (2013). Protein phosphatase 2A regulatory subunits perform distinct functional roles in the maize pathogen *Fusarium verticillioides*. *Mol. Plant Pathol.* 14 518–529. 10.1111/mpp.12023 23452277 PMC6638791

[B45] Springer M. L. (1993). Genetic control of fungal differentiation the 3 sporulation pathways of *Neurospora crassa*. *Bioessays* 15 365–374. 10.1002/bies.950150602 8357339

[B46] St-Denis N. Gupta G. D. Lin Z. Y. Gonzalez-Badillo1 B. Veri A. O. Knight J. D. R. (2016). Phenotypic and interaction profiling of the human phosphatases identifies diverse mitotic regulators. *Cell Rep.* 17 2488–2501. 10.1016/j.celrep.2016.10.078 27880917

[B47] Steele G. C. Trinci A. P. J. (1977). Effect of temperature and temperature shifts on growth and branching of a wild type and a temperature sensitive colonial mutant (Cot 1) of *Neurospora crassa*. *Arch. Microbiol.* 113 43–48. 10.1007/BF00428578 142456

[B48] Stegert M. R. Hergovich A. Tamaskovic R. Bichsel S. J. Hemmings B. A. (2005). Regulation of NDR protein kinase by hydrophobic motif phosphorylation mediated by the mammalian Ste20-like kinase MST3. *Mol. Cell. Biol.* 25 11019–11029. 10.1128/MCB.25.2f4.11019-11029.2005 16314523 PMC1316964

[B49] Tamaskovic R. Bichsel S. J. Rogniaux H. Stegert M. R. Hemmings B. A. (2003). Mechanism of Ca2+-mediated regulation of NDR protein kinase through autophosphorylation and phosphorylation by an upstream kinase. *J. Biol. Chem.* 278 6710–6718. 10.1074/jbc.M210590200 12493777

[B50] Turra D. Di Pietro A. (2015). Chemotropic sensing in fungus-plant interactions. *Curr. Opin. Plant Biol.* 26 135–140. 10.1016/j.pbi.2015.07.004 26247120

[B51] Virshup D. M. Shenolikar S. (2009). From promiscuity to precision: protein phosphatases get a makeover. *Mol. Cell* 33 537–545. 10.1016/j.molcel.2009.02.015 19285938

[B52] Widau R. C. Jin Y. Dixon S. A. Wadzinski B. E. Gallagher P. J. (2010). Protein phosphatase 2A (PP2A) holoenzymes regulate death-associated protein kinase (DAPK) in ceramide-induced anoikis. *J. Biol. Chem.* 285 13827–13838. 10.1074/jbc.M109.085076 20220139 PMC2859546

[B53] Xu Y. Xing Y. Chen Y. Chao Y. Lin Z. Fan E. (2006). Structure of the protein phosphatase 2A holoenzyme. *Cell* 127 1239–1251. 10.1016/j.cell.2006.11.033 17174897

[B54] Yang Y. H. He Q. Cheng P. Wrage P. Yarden O. Liu Y. (2004). Distinct roles for PP1 and PP2A in the *Neurospora* circadian clock. *Genes Dev.* 18 255–260. 10.1101/gad.1152604 14871927 PMC338279

[B55] Yarden O. Plamann M. Ebbole D. J. Yanofsky C. (1992). *cot-1*, a gene required for hyphal elongation in *Neurospora crassa*, encodes a protein kinase. *EMBO J.* 11 2159–2166. 1534751 10.1002/j.1460-2075.1992.tb05275.x PMC556683

[B56] Yatzkan E. Szoor B. Feher Z. Dombradi V. Yarden O. (1998). Protein phosphatase 2A is involved in hyphal growth of *Neurospora crassa*. *Mol. Gen. Genet.* 259 523–531. 10.1007/s004380050844 9790584

[B57] Yatzkan E. Yarden O. (1995). Inactivation of a single-2A phosphoprotein phosphatase is lethal in *Neurospora crassa*. *Curr. Genet.* 28 458–466. 10.1007/BF00310816 8575020

[B58] Yatzkan E. Yarden O. (1999). The B regulatory subunit of protein phosphatase 2A is required for completion of macrocondiation and other developmental processes in *Neurospora crassa*. *Mol. Microbiol.* 31 197–209. 10.1046/j.1365-2958.1999.01161.x 9987122

[B59] Zhong G. W. Jiang P. Qiao W. R. Zhang Y. W. Wei W. F. Lu L. (2014). Protein phosphatase 2A (PP2A) regulatory subunits ParA and PabA orchestrate septation and conidiation and are essential for PP2A activity in *Aspergillus nidulans*. *Eukaryot. Cell* 13 1494–1506. 10.1128/EC.00201-14 25280816 PMC4248680

[B60] Ziv C. Feldman D. Aharoni-Kats L. Chen S. Liu Y. Yarden O. (2013). The N-terminal region of the *Neurospora* NDR kinase COT1 regulates morphology via its interactions with MOB2A/B. *Mol. Microbiol.* 90 383–399. 10.1111/mmi.12371 23962317 PMC4603829

[B61] Ziv C. Kra-Oz G. Gorovits R. Maerz S. Seiler S. Yarden O. (2009). Cell elongation and branching are regulated by differential phosphorylation states of the nuclear Dbf2-related kinase COT1 in *Neurospora crassa*. *Mol. Microbiol.* 74 974–989. 10.1111/j.1365-2958.2009.06911.x 19818014

[B62] Ziv C. Yarden O. (2010). Gene silencing for functional analysis: assessing RNAi as a tool for manipulation of gene expression. *Methods Mol. Biol.* 638 77–100. 10.1007/978-1-60761-611-5-6 20238262

[B63] Zolnierowicz S. (2000). Type 2A protein phosphatase, the complex regulator of numerous signaling pathways. *Biochem. Pharmacol.* 60 1225–1235. 10.1016/S0006-2952(00)00424-X 11007961

