# Supplementary material for: The Neurospora crassa PP2A Regulatory Subunits RGB1 and B56 Are Required for Proper Growth and Development and Interact with the NDR Kinase COT1
Source: Front Microbiol. 2017 Sep 5;8:1694. doi: 10.3389/fmicb.2017.01694 (PMC5591878; doi:10.3389/fmicb.2017.01694)
Supplement: Supplementary file 1 [file Table_1.PDF]

*Supplementary Material*

# The *Neurospora crassa* PP2A Regulatory Subunits RGB1 and B56 are Required for Proper Growth and Development and Interact with the NDR Kinase COT1

*Hila Shomin-Levi and Oded Yarden*

*** Correspondence:** Corresponding Author: oded.yarden@mail.huji.ac.il

**Supplementary Table S1: Identification of COT1-PPH1-interacting proteins by LC-MS.**

Data from two independent biological replicates were used for protein-protein interaction identification. The identified proteins were filtered against a WT as a control. Only proteins identified in two independent experiments and absent from a control data set are shown. Protein coverage by peptides and the number of identified unique peptides is displayed. Semi quantitation was done by calculating the peak area of each peptide. Area of the protein is the average of the three most intense peptides from each protein

| Detected protein | NCU | Description | Σ Coverage | Σ Unique Peptides | WT: Area | COT1: Area | PPH1: Area |
|---|---|---|---|---|---|---|---|
| COT1 | NCU07296 | serine/threonine kinase<br><br>(Yarden et al., 1992) | 61.04 | 40 | 0.000E0 | 1.778E9 | 3.245E7 |
| B56 | NCU03786 | serine/threonine protein phosphatase 2A | 17.91 | 10 | 0.000E0 | 6.503E6 | 2.002E8 |
| PPH1 | NCU06630 | serine/threonine-protein phosphatase PP2A catalytic subunit (Yatzkan et al., 1998) | 42.81 | 11 | 0.000E0 | 1.995E7 | 4.164E8 |
| GUL1 | NCU01197 | related to SSD1 protein (Herold and Yarden, 2017) | 5.74 | 6 | 0.000E0 | 1.077E7 | 9.850E7 |
| PMR3 | NCU01669 | related to protein arginine N-methyltransferase 3 (Feldman et al., 2013) | 10.56 | 4 | 0.000E0 | 0.000E0 | 7.455E7 |
| MOB2B | NCU07460 | protein kinase activator (Ziv et al 2013) | 8.91 | 2 | 0.000E0 | 3.794E6 | 4.747E6 |
